# Transcriptome analysis of osmotic-responsive genes in ABA-dependent and -independent pathways in wheat (*Triticum aestivum* L.) roots

**DOI:** 10.7717/peerj.6519

**Published:** 2019-03-05

**Authors:** Chunxi Li, Wenli Zhang, Meng Yuan, Lina Jiang, Bo Sun, Daijing Zhang, Yun Shao, Anqi Liu, Xueqing Liu, Jianhui Ma

**Affiliations:** College of Life Science, Henan Normal University, Xinxiang, The People's Republic of China

**Keywords:** *Triticum aestivum* L., Osmotic stress, Abscisic acid, Transcriptomic

## Abstract

Bread wheat is one of the most important crops in the world. However, osmotic stress significantly inhibits wheat growth and development, and reduces crop yield and quality. Plants respond to osmotic stress mainly through abscisic acid (ABA)-dependent and -independent pathways. In this study, root transcriptome profiles of wheat seedlings exposed to osmotic stress and exogenous ABA were analysed to identify osmotic-responsive genes belonging to the ABA-dependent or -independent pathways. We found that osmotic stress promoted proline biosynthesis in the ABA-dependent pathway, and trehalose biosynthesis is likely promoted among soluble sugars to maintain protein bioactivity under osmotic stress. In wheat roots subjected to osmotic stress, calcium ions, and glutathione exert their functions mainly through calcium-binding protein (CaM/CML) and glutathione-*S*-transferase, respectively, depending on both pathways. In addition, a complex relationship among phytohormones signal transduction was observed in response to osmotic stress. The findings of this study deepen our understanding of the molecular mechanisms of osmotic-stress resistance, and provide several candidate osmotic-responsive genes for further study.

## Introduction

Osmotic stress refers to insufficient water availability, which limits plant growth, and mainly results from drought or excessive salt in water (Zhu et al., 1997). Bread wheat, one of the most important cereal crops, is consumed as a main food source by 40% of the world’s population (Shewry, 2009). However, abiotic stress, especially osmotic stress, has dramatically impacted wheat production. For example, Zhang et al. (2013) analysed data from 264 agricultural meteorological observation stations in China from 1991 to 2009, and found that water deficit, resulting from drought, accounted for 79.21% of all meteorological disasters. To further improve osmotic tolerance in wheat, it is necessary to understand the molecular mechanisms that are invoked in response to osmotic stress.

Under conditions of osmotic stress, abscisic acid (ABA) is generally considered as a stress signalling hormone, and expression of stress-responsive genes in plants is primarily regulated by ABA-dependent and -independent pathways (Yoshida, Mogami & Yamaguchi-Shinozaki, 2014; Agarwal & Jha, 2010). The ABA-dependent pathway is central to osmotic-stress responses in plants. Previous studies have identified that pyrabactin resistance1/PYR1-like/regulatory components of ABA receptors (PYR/PYL/RCAR) and protein phosphatases type-2C (PP2C) serve as ABA co-receptors (Park et al., 2009; Ma et al., 2009). ABA binds to PYR/PYL/RCAR and inhibits *PP2C*. Consequently, SNF1-related protein kinases 2 (SnRK2s) are activated by phosphorylation at multiple sites under drought stress, a process that regulates the expression levels of downstream genes (Nishimura et al., 2010). The transcription factors ABRE-binding protein/ABRE-binding factors (*AREB/ABFs*), such as *AREB1*, *AREB2*, *ABF1*, *ABF2,* and *ABF3*, are induced by osmotic stress, and *AREB/ABFs* are positively regulated by SnRK2s in the ABA-dependent pathway. Moreover, overexpression of *AREB/ABFs* may increase drought tolerance via the ABA-dependent pathway (Fujita et al., 2005; Kim et al., 2004; Furihata et al., 2006). Nevertheless, as many drought-inducible genes do not respond to ABA treatment, there is likely another ABA-independent mechanism regulating osmotic-stress response. Dehydration-responsive element/C-repeat-binding protein 2 (*DREB2*) is a member of the *AP2/ERF* family and a key transcription factor in the ABA-independent pathway. *DREB2A* and *DREB2B* are highly induced by osmotic stress and significantly enhance resistance to osmotic stress in plants. Under suitable growth conditions, the DREB2A protein is tightly regulated by ubiquitin E3 ligases (Sakuma et al., 2006). *DREB2A* activity is also repressed by *GROWTH-REGULATION FACTOR7* (*GRF7*), which may bind to the *DREB2A* promoter to repress *DREB* expression (Kim et al., 2012). Overall, genes induced by both osmotic stress and ABA may participate in the ABA-dependent pathway, whereas those induced only by osmotic stress are likely involved in the ABA-independent pathway.

High-throughput sequencing is developing rapidly in recent years, and the results can provide an overview of gene expression in a given system to better understand the molecular mechanisms. For instance, Zeng et al. (2016) applied transcriptome analysis to reveal drought-responsive genes in Tibetan hulless barley, and found that majority of up-regulated genes under a low relative soil moisture content are involved in ABA-dependent and -independent pathways. Ma et al. (2017) also used transcriptome analyses to characterise wheat responses to drought stress under field condition, and reported that the drought-responsive genes are largely involved in floral development, photosynthesis, and stomatal movement. Accordingly, we performed a proteomic profile analysis of wheat roots exposed to osmotic stress and found that the glutathione (GSH) system plays an important role in this response (Ma et al., 2016). To understand gene expression in ABA-dependent and -independent pathways in response to drought stress, *Arabidopsis thaliana* plants treated with drought, ABA and corresponding controls were sampled for RNA sequencing (Liu et al., 2018), and interactions between ABA-dependent and -independent pathways were shown to be enriched. These findings provide an overview of molecular mechanism underlying responses to abiotic stress.

With regard to molecular function analysis, previous studies have revealed that certain wheat genes like *TabHLH1*, *TaWRKY33*, *TaAIDFa*, and others, can be induced by both osmotic stress and ABA. Overexpression of these genes increases drought tolerance, which indicated that they may confer drought resistance via the ABA-dependent pathway (Yang et al., 2016; He et al., 2016; Xu et al., 2008). Although some transcriptome and proteomic profile studies focusing on osmotic stress in wheat have been performed, we were not able to integrally classify osmotic-responsive genes involved in ABA-dependent or -independent pathways. In this study, we used transcriptome profiles to identify osmotic-responsive genes participating in the ABA-dependent or -independent pathways. Furthermore, the biological functions of these genes were analysed to understand the mechanism of osmotic-stress response in wheat roots.

## Materials and Methods

### Plant material and growth conditions

The winter wheat cultivar Aikang 58, the production of which nearly accounts for 10% of total wheat production in China, was selected for this study. Wheat seeds were sterilised in a 0.1% w/v HgCl_2_ solution for 8 min, washed three times with distilled water and immersed in water at 18 °C in the dark. Seeds of similar size were selected and germinated on wet filter paper in aseptic Petri dishes (diameter 15 cm). After germination, the seedlings were cultivated in Hoagland solution until the two-leaf stage in a growth chamber at 18 ± 1 °C with a relative humidity of 75% and a photoperiod of 12 h illumination at 300 μmol/m^2^/s^1^ light intensity (Li et al., 2013).

At the two-leaf stage, the wheat seedlings were subjected to treatments with 15% PEG-6000 for osmotic stress and 100 μM ABA in Hoagland solution; a control (CK) was also included. After 3, 6, 12, 18, and 24 h, roots samples were rinsed with distilled water, harvested, and stored at −80 °C.

### Total RNA extraction and transcriptome sequencing

Root samples were selected at 3 and 24 h. Total RNA of 18 samples (three replicates per treatment: CK_3 h, ABA_3 h, PEG_3 h, CK_24 h, ABA_24 h, and PEG_24 h) was extracted using TRIzol reagent (Takara Bio Inc., Kusatsu, Shiga, Japan) according to the manufacturer’s instructions for transcriptome sequencing.

A total of three μg RNA per sample was used to build cDNA libraries with a NEBNext^®^ Ultra™ RNA Library Prep Kit for Illumina^®^ (NEB, Ipswich, MA, USA) according to the manufacturer’s instructions. The cDNA library was sequenced using the Illumina sequencing platform (HiSeq-PE150). Raw reads were first processed through in-house Perl scripts. Adapter sequences, low-quality reads, and reads containing more than 10% ambiguous ‘N’ bases were removed. The Q20, Q30, and GC contents of the remaining clean reads were calculated. The reference genome was downloaded directly from the genome website (Clavijo et al., 2017). An index of the reference genome was generated, and paired-end clean reads were aligned to the reference genome using Bowtie v 2.2.3 and Top Hat v. 2.0.12, respectively. The raw transcriptome reads are available at NCBI (https://www.ncbi.nlm.nih.gov/) under Bioproject ID PRJNA464413. The SRA accession number is SRP145238.

### Measurement of root activity, glutathione-*S*-transferase (GST) activity, and proline, soluble sugar, and ABA contents

Root activity was determined using triphenyl tetrazolium chloride (TTC) method (Luo, Zhang & Zhang, 2016). Briefly, 0.5 g roots were added to tubes containing five mL of 0.4% (w/v) TTC and five mL of phosphate buffer (0.067 M, PH 6.89). After reaction at 37 °C for 3 h in dark, the reaction was terminated by adding two ml of sulphuric acid (one mol/L). The roots were subsequently taken out and placed in another tube containing 10 ml methanol for triphenylformazan (TPF) extraction. After incubation at 37 °C for 7 h, the optical density values of supernatant were recorded by an ultraviolet spectrophotometer at 485 nm, and were used to calculate root activity via TPF concentration. To measure GST activity, 0.25 g fresh roots was milled in two mL precooled phosphate buffer (0.1 M, pH 6.5) and ground at low temperature. The mixture was centrifuged at 10,000 rpm for 15 min, and the supernatant was used for GST activity determination according to the method of Zhang & Ying (2008). The proline content was determined in the wheat roots at various treatment stages using the method of Demiral & Türkan (2005). The soluble sugar content was assessed with the anthrone reagent and a Bausch + Lomb Spectrophotometer (Hui et al., 2012). Endogenous ABA was extracted and purified from 0.1 g roots using a method previously described by Vilaró, Canela-Xandri & Canela (2006). The filtered samples were measured using an HPLC (Waters 600; Waters Corporation, Milford, MA, USA) fitted with a photodiode array detector and a C18 column (4.6 mm × 150 mm; five μm) following the method of Wang et al. (2008) with some modifications. Compound separation was evaluated with a mobile phase of aqueous methanol: 0.2% formic acid (20:80, v/v) at a flow rate of 0.5 mL min^−1^. The extract injection volume per sample was 10 μL, the detection wavelength was 254 nm, and the column oven temperature was 35 °C. All samples were purified through 0.45-μm filter membranes before being injected. ABA concentrations were determined from the peak areas based on standard curve.

### Differentially expressed genes and pathways analysis

Fragments mapped onto full-length cDNA sequences were counted by HTSeq v0.6.1. The expected number of fragments per kilobase of transcript sequence per millions base pairs sequenced (FPKM) was used to eliminate the effects of differential sequencing depth and gene length per read. FPKM values were calculated to normalise and determine the expression values of each gene. Differentially expressed genes (DEGs) were identified by the parameters of fold change ≥ 4. Kyoto Encyclopaedia of Genes and Genomes (KEGG) enrichment analyses were conducted using KOBAS software.

### Quantitative real-time PCR assay

The transcriptome assay was validated by quantitative real-time PCR (qRT-PCR). The primer sequences were designed by Oligo7, as listed in Supplementary File 1. Total RNA samples were extracted from roots of each treatment. Any residual DNA was removed with gDNA Eraser at room temperature. First-strand cDNA synthesis was performed with 15 μg RNA. The qRT-PCR amplification was performed using ChamQTM SYBR^®^ qPCR Master Mix (Vazyme Biotech Co. Ltd., Nanjing City, China) on a LightCycler^®^ 96 system, as follows: one cycle of 95 °C for 30 s; 35 cycles of 95 °C for 10 s; and 60 °C for 30 s. The relative quantitative method (2^−ΔΔ*CT*^) was used to calculate the expression levels of target genes (Livak & Schmittgen, 2001).

## Results

### Effects of osmotic stress and exogenous ABA on wheat seedlings

**Figure 1:**
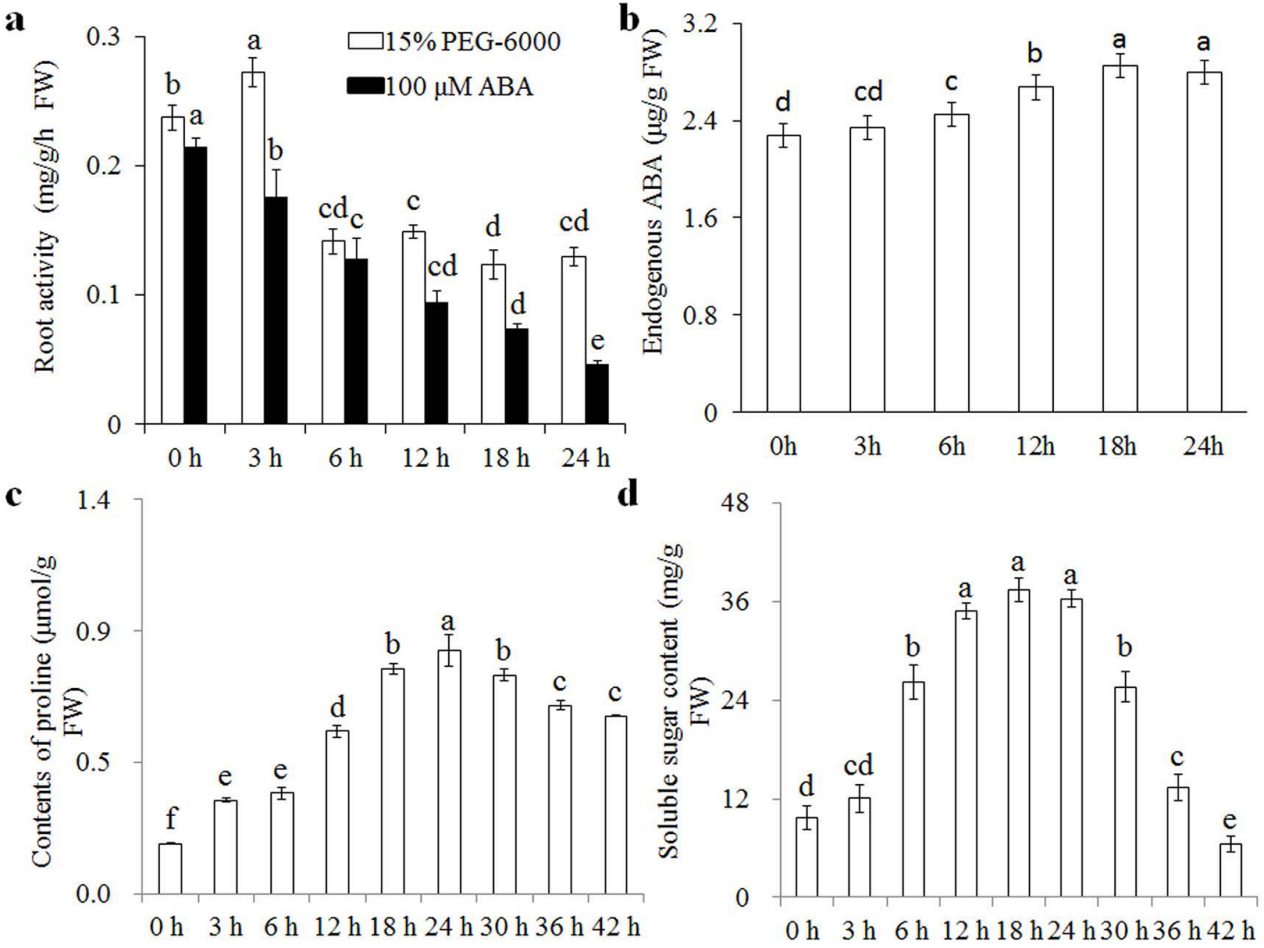
Root activity under osmotic stress and 100 μM ABA treatment. Root activity (A) under osmotic stress and 100 μM ABA treatment at 0, 3, 6, 12, 18, and 24 h, and changes in endogenous ABA (B), proline (C) and soluble sugar (D) contents under osmotic stress at various time points. Each experiment was carried out with three biological replicates. Values are means ± SEs. Analysis of variance (ANOVA) was performed, and least significant difference (LSD) was used for multiple comparisons. Lowercase letters indicate significant differences at 0.05 level.

Wheat seedlings were cultured in Hoagland solution with 15% PEG-6000 or 100 μM ABA for different times (0, 3, 6, 12, 18, and 24 h), and root samples were collected to measure root activity, endogenous ABA, proline, and soluble sugar contents. Root activity increased significantly after 3 h of osmotic stress, but levelled off and stabilised after 6 h (Fig. 1A). The content of endogenous ABA increased gradually after 3 h of osmotic stress and was stabilised after 18 h. Proline and soluble sugar contents increased after 3 h of osmotic stress, and reached peak values after 24 h (Figs. 1B–1D). Root activity was also measured to analyse the effects of exogenous ABA, showing a gradual decrease with increasing treatment duration. We therefore speculated that wheat seedlings have a rapid response to osmotic stress in the short term (3 h) and another response in the relative long term (24 h). Therefore, we selected the 3 and 24 h treatments to examine osmotic-stress response in wheat seedlings.

### Transcriptome sequencing and de novo assembly

Eighteen cDNA libraries (three replicates per treatment: CK_3 h, ABA_3 h, PEG_3 h, CK_24 h, ABA_24 h, and PEG_24 h) were constructed and the RNA-seq libraries were sequenced using the Illumina sequencing platform (HiSeq-PE150). A total of 1,352,604,888 clean reads were obtained for the six testing samples after removing low-quality reads and adapter sequences (Table 1). The average base sequencing error rate was 0.024%, and the average G and C bases proportion was 55.77%. Q20 and Q30 were 94.98% and 87.81%, respectively. Therefore, all libraries were of high quality. All clean reads were pooled, and Trinity pv. 2012-10-05 was used to generate a de novo assembly. Finally, 128,679 putative genes were obtained. Correlation of gene expression among the three biological replicates was calculated with correlation coefficients (R^2^) of 0.971, 0.979, 0.942, 0.983, 0.974, and 0.975 for CK_3 h, PEG_3 h, ABA_3 h, CK_24 h, PEG_24 h, and ABA_24 h, respectively. These results indicate high correlations among the replicates. Six genes were randomly selected for qRT-PCR verification of the transcriptome results. We found that the transcriptome data were supported by the qRT-PCR results (Supplementary File 1), and could thus be applied in the subsequent analysis.

**Table 1:** Summary of transcriptome sequencing and correlations among samples in the same treatment.

Samples	Raw reads	Clean reads	Error rate	Q20 (%)	Q30 (%)	GC (%)	Total mapped	Multiple mapped	Uniquely mapped	Coefficients (R2)
CK_3h	218,490,438	208,789,868	0.02	94.95	87.55	54.92	192,067,747 (91.99%)	6,822,473 (3.27%)	185,245,274 (88.72%)	0.971
PEG_3h	271,963,080	261,796,530	0.02	95.46	89.17	55.80	236,570,203 (90.48%)	8,018,740 (3.04%)	228,551,463 (87.44%)	0.979
ABA_3h	265,514,198	255,256,216	0.02	95.17	88.50	56.51	233,948,009 (91.65%)	7,710,363 (3.02%)	226,237,646 (88.63%)	0.942
CK_24h	225,590,634	214,213,292	0.02	94.79	87.23	55.43	195,066,383 (91.06%)	6,885,508 (3.22%)	188,180,875 (87.85%)	0.983
PEG_24h	215,594,980	205,732,464	0.02	94.77	87.25	55.40	186,889,621 (90.83%)	6,375,284 (3.10%)	180,514,337 (87.74%)	0.974
ABA_24h	217,446,650	207,191,838	0.03	94.73	87.17	56.56	189,146,146 (91.3%)	6,235,028 (3.01%)	182,911,118 (88.29%)	0.975

### Analysis of DEGs in ABA-dependent and -independent pathways

**Figure 2:**
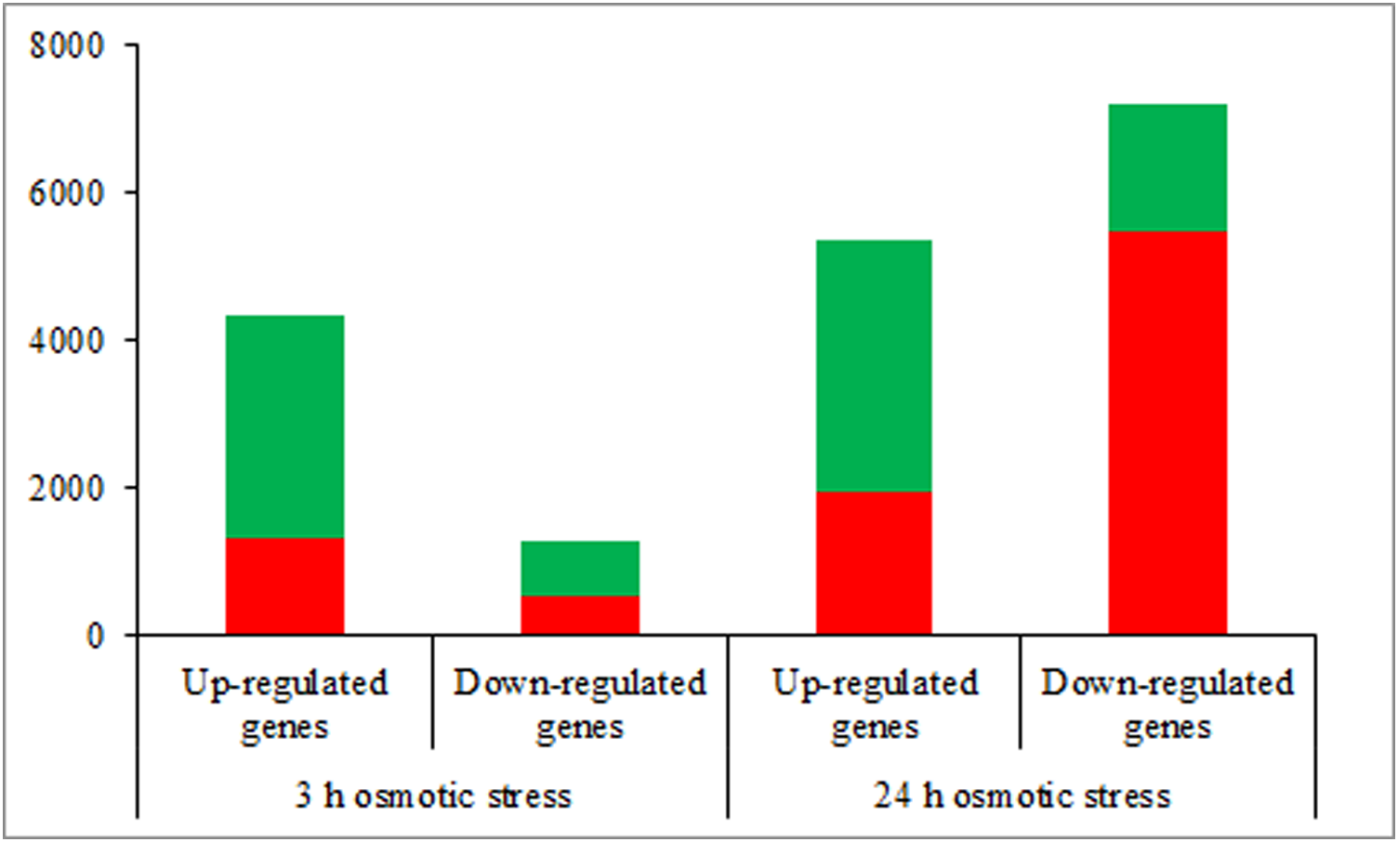
The number of differentially expressed genes in ABA-dependent and -independent pathways in response to osmotic stress for 3 and 24 h, respectively. The histograms in red represent genes involved in ABA-dependent pathway, and the histograms in green represent genes involved in ABA-independent pathway.

**Figure 3:**
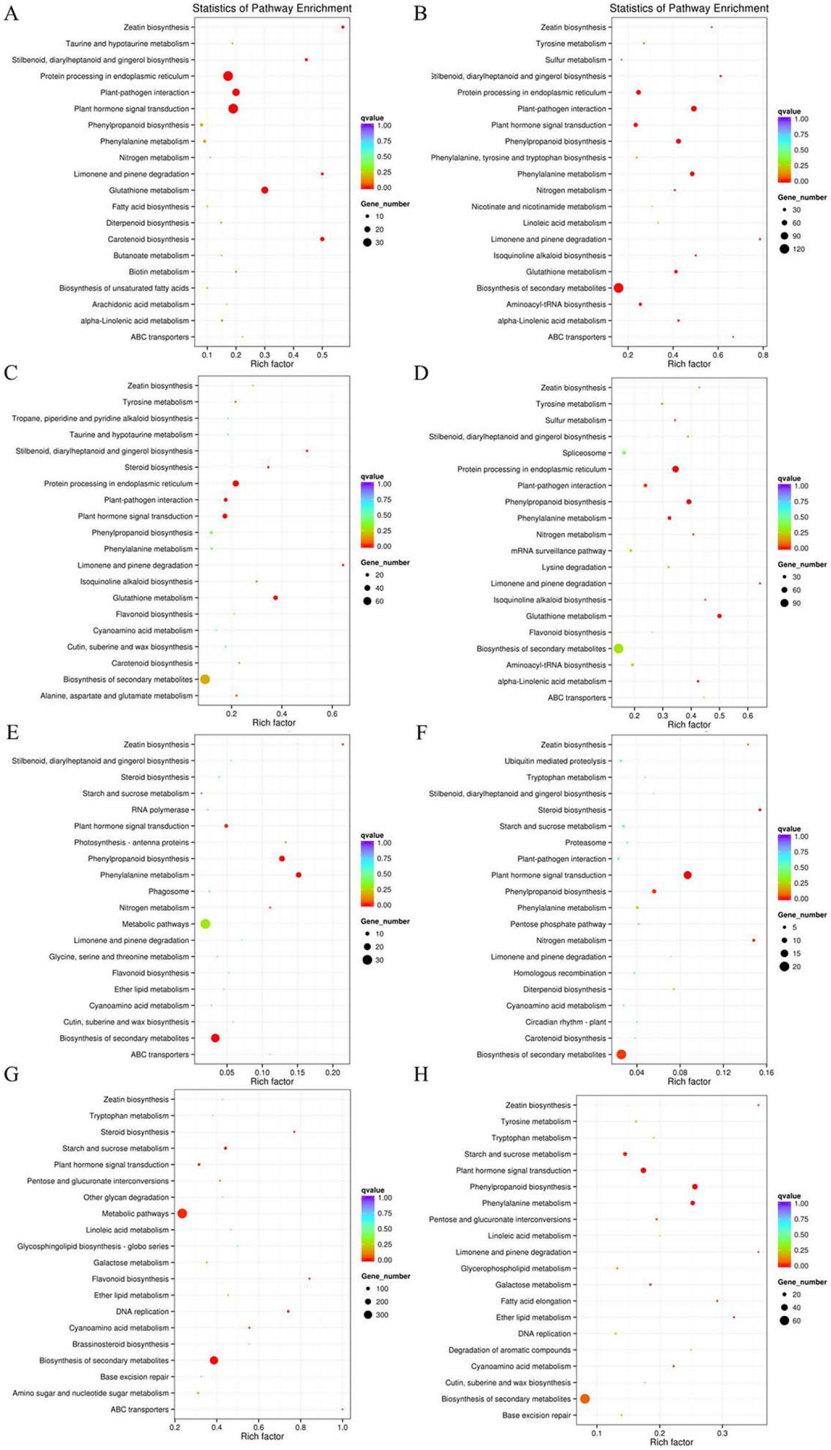
Pathway enrichment under osmotic stress for 3 and 24 h. DEGs were classified into eight groups. Pathway enrichment of these up-regulated DEGs involved in ABA-dependent and -independent pathways after 3 and 24 h of osmotic stress is shown in (A–D), respectively. Pathway enrichment of these down-regulated DEGs involved in ABA-dependent and -independent pathways after 3 and 24 h of osmotic stress is shown in (E–H), respectively.

According to the DEGs selection parameters applied, 4,323 up-regulated and 1,270 down-regulated genes were identified for the 3 h osmotic stress treatment, and 5,364 up-regulated and seven down-regulated genes were identified for the 24 h osmotic stress treatment. Function annotations for all DEGs are provided in Supplementary File 2. Of these, 1,889 up-regulated and 528 down-regulated genes continuously responded to osmotic stress. In previous studies, it was assumed that DEGs, induced by osmotic stress but not responding to ABA treatment, participate in the ABA-independent pathway, and those, induced by both osmotic stress and exogenous ABA, are involved in the ABA-dependent pathway (Shinozaki & Yamaguchi-Shinozaki, 2007). The DEGs identified in this study were further classified into eight groups in the ABA-dependent or -independent pathways, as shown in Fig. 2. KEGG analysis was performed for the genes in each group (Fig. 3), revealing some important pathways, enzymes, and transcription factors.

#### Differentially expressed genes involved in ABA biosynthesis and signalling

**Figure 4:**
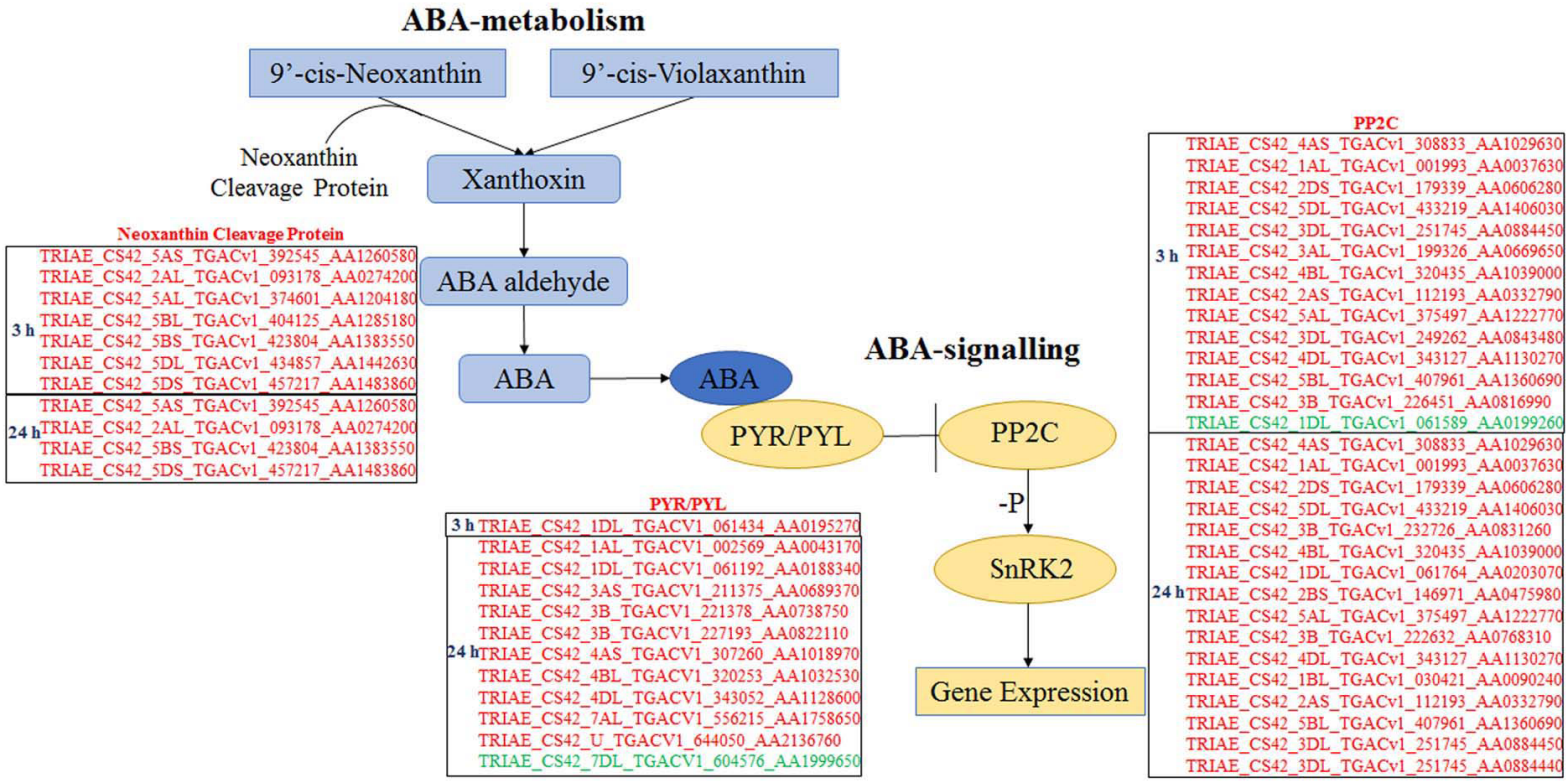
Differentially expressed genes participating in ABA biosynthesis and ABA signalling selected by Mapman. Genes in red are involved in ABA-dependent pathway, and genes in green colour are involved in ABA-independent pathway.

All DEGs were analysed using software Mapman (Thimm et al., 2004), and those participating in ABA biosynthesis and signalling are listed in Fig. 4. Seven and four genes, which encode neoxanthin cleavage protein and participate in ABA biosynthesis, were all up-regulated at 3 and 24 h, respectively, under ABA and osmotic stress. It indicated that all these genes are involved in the ABA-dependent pathway. Twelve *PYR/PYLs* (ABA co-receptors) were found to be down-regulated under osmotic stress; of these, 11 were regulated by both ABA and osmotic stress, whereas one was only regulated by osmotic stress. Twenty *PP2Cs* were found to be up-regulated under osmotic stress, 10 of which were up-regulated continuously for 3 to 24 h osmotic stress; of these, 19 were induced by ABA and osmotic stress and one was just induced by osmotic stress. Based on these results, the majority of core genes in ABA biosynthesis and signalling participate in the ABA-dependent pathway, and the findings also indicate that this DEG classification system is reasonable.

#### Calcium ions signalling

**Figure 5:**
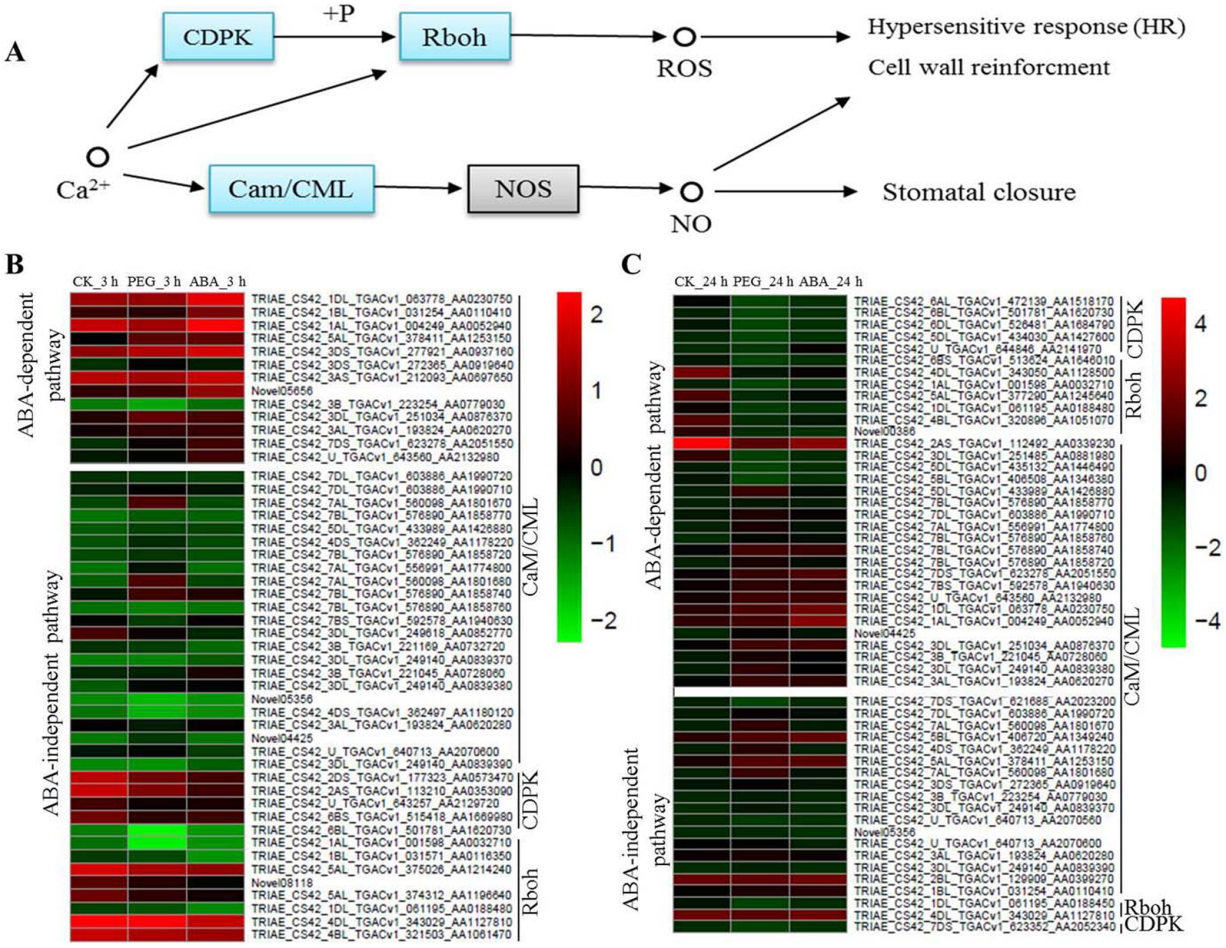
Calcium signalling in response to osmotic stress and expression levels of associated genes at 3 and 24 h of osmotic stress. Calcium mainly plays its role through *Cam/CML*, *CDPK* and *Rboh* responding to osmotic stress in plants (A). Expression levels of genes associated with osmotic stress in the ABA-independent and -dependent pathways at 3 h (B) and 24 h (C).

Calcium is a multifunctional secondary messenger. It plays an important role in signal transduction. Cytoplasmic Ca^2+^ concentrations change under osmotic stress, further causing responses by *CaM/CML*, Calcium-dependent protein kinases (*CDPK*) and Respiratory burst oxidase homologue (*Rboh*) protein (Fig. 5A). In the present study, many osmotic-responsive genes, involved in calcium ions signalling, were found. Among these DEGs, 36 and 33 *CaM/CMLs* were up-regulated after 3 and 24 h of osmotic stress respectively, and only 4 *CaM/CMLs* were down-regulated after 24 h of osmotic stress. More than ten *CaM/CMLs* in the ABA-dependent or -independent pathways were found after 3 and 24 h of osmotic stress respectively. It is interesting that majority of *CDPKs* and *Rbohs* were up-regulated in ABA-independent pathway at 3 h, whereas majority of them were down-regulated in ABA-dependent pathway at 24 h. Calcium ions may promote the phosphorylation of CDPK to regulate Rboh proteins, resulting in the generation of reactive oxygen species (ROS). We found that expression of *CDPKs* and *Rbohs* was suppressed with increasing ABA content. Therefore, ABA appears to be a negative regulatory factor of *CDPKs* and *Rbohs*, and calcium ions should mainly exert their effects via *CaM/CMLs* to regulate osmotic response in wheat roots.

#### Glutathione metabolism

**Figure 6:**
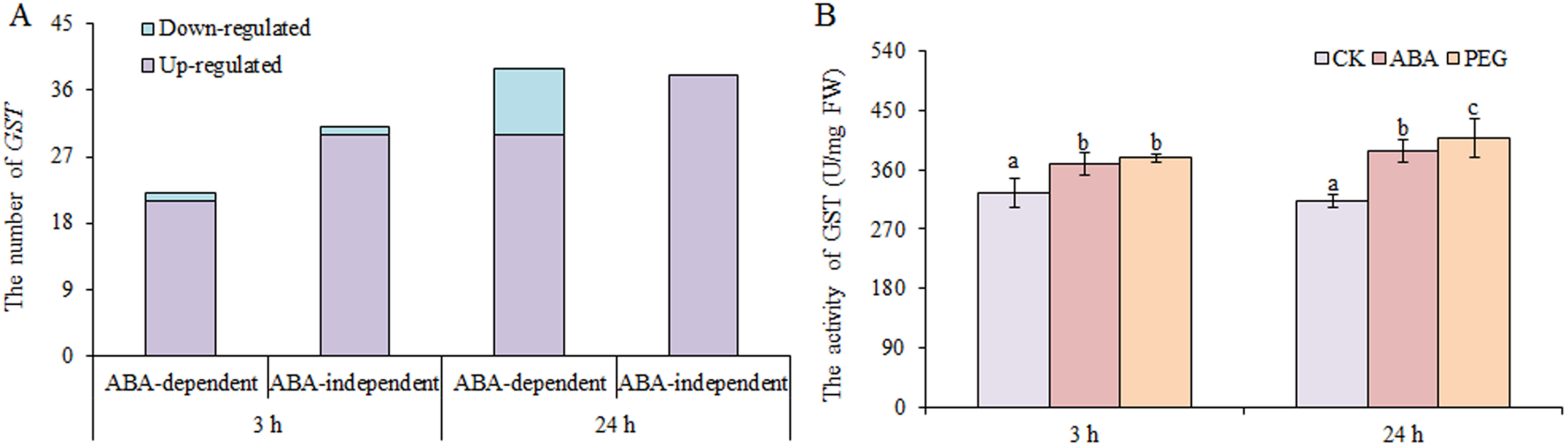
Number of *GSTs* (A) and GST activity (B) in wheat roots in response to ABA and osmotic stress. Three biological replicates were used per treatment. Values are means ± SEs. ANOVA was performed, and LSD was used for multiple comparisons. Lowercase letters indicate significant differences at the 0.05 level.

Glutathione antioxidant and detoxification activities in plants largely occur through GSH-GSSH (glutathione disulphide) cycle, which is dependent on GSH reductase and GSH peroxidase, and GSTs (Ma et al., 2016). We found that only two DEGs encoding GSH peroxidases and one DEGs encoding GSH reductase were up-regulated under osmotic stress. However, many *GSTs* were up-regulated under osmotic stress, including 51 at 3 h and 68 at 24 h, nearly half of which were involved in ABA-dependent or -independent pathways at both time points (Fig. 6). In contrast, only two *GSTs* and nine *GSTs* were down-regulated under osmotic stress at 3 and 24 h, respectively. GST activity was also measured, and the results showed that GST activity increased significantly under ABA and PEG treatments at 3 and 24 h, respectively (Fig. 6B). Therefore, we speculate that the role of GSH is mainly exerted through GST in wheat roots under osmotic stress, as simultaneously regulated by ABA-dependent and -independent pathways.

#### Phytohormones signal transduction

Many DEGs are involved in phytohormones signal transduction. In our study, 36 (22 down- and 14 up-regulated DEGs) and 69 DEGs (55 down- and 14 up-regulated DEGs) involved in auxin signal transduction at 3 and 24 h respectively, and the majority of these genes were down-regulated with the increasing of treatment duration. It had been reported that osmotic stress can regulate auxin transporter levels to reduce root auxin concentrations in a manner dependent on the ABA-dependent pathway (Rowe et al., 2016). We also found that genes encoding auxin transporter protein 1, which is required for auxin influx into cells (Swarup et al., 2001; Yang et al., 2006), were all down-regulated in ABA-dependent pathway at 24 h; this would further affect the expression of downstream gene, such as those encoding auxin responsive and induced proteins (GH3, SAUR, and AUX/IAA), to impact plant growth. In the cytokinin signal transduction, five *Arabidopsis response regulators* (*ARRs*), which display increased expression under cytokinin treatment (Brandstatter & Kieber, 1998), were down-regulated in the ABA-independent pathway. This result indicates that the cytokinin content should be decreased under osmotic stress to affect cell division via ARR. As *ARR* might be negatively regulated by ethylene (Rowe et al., 2016), and DEGs, encoding ethylene receptor (ETR), ethylene-insensitive protein (EIN), and ethylene-responsive transcription factor (ERF) in ethylene signal transduction were all up-regulated. This result suggests that the ethylene content should increase via ABA-dependent and -independent pathways. In jasmonic acid signal transduction, 15 up-regulated *JAZs* and only one down-regulated *JAZ* were observed under osmotic stress, which is regulated by both ABA-dependent and -independent pathways. Therefore, it is important to investigate relationships among phytohormones responding to osmotic stress in plants.

#### Metabolism of low-molecular weight compounds

The content of low-molecular weight compounds, such as proline and soluble sugars, should increase to enhance osmotic potential under osmotic stress, and DEGs involved in the metabolism of these compounds were also found. In plants, proline could be synthesised via glutamate and ornithine pathways, whereas it may be degraded and transformed by proline dehydrogenase (ProDH) and prolyl 4-hydroxylase (ProLH). Pyrroline-5-carboxylate synthetase (P5CS), a key enzyme in proline biosynthesis, could be induced by osmotic stress (Peng, Lu & Verma, 1996). In the present study, the up-regulation of *P5CSs* was found at 3 and 24 h, and the down-regulation of *ProDH* and *ProLH* were found at 24 h in ABA-dependent pathway. This result indicates that ABA induced proline synthesised via P5CS, and reduced proline degradation via ProDH and ProLH to increase osmotic potential in wheat roots under osmotic stress. With regard to carbohydrate metabolism, DEGs, encoding amylase for hydrolysis of starch, were up-regulated. It is interesting that four and eight genes involved in trehalose biosynthesis were up-regulated at 3 and 24 h, respectively, and that the majority of these genes depend on the ABA-independent pathway. We also found that the content of soluble sugars increased significantly under osmotic stress, whilst it is important to determine which kind of soluble sugars play a major role in future studies.

#### Transcription factor analysis

**Figure 7:**
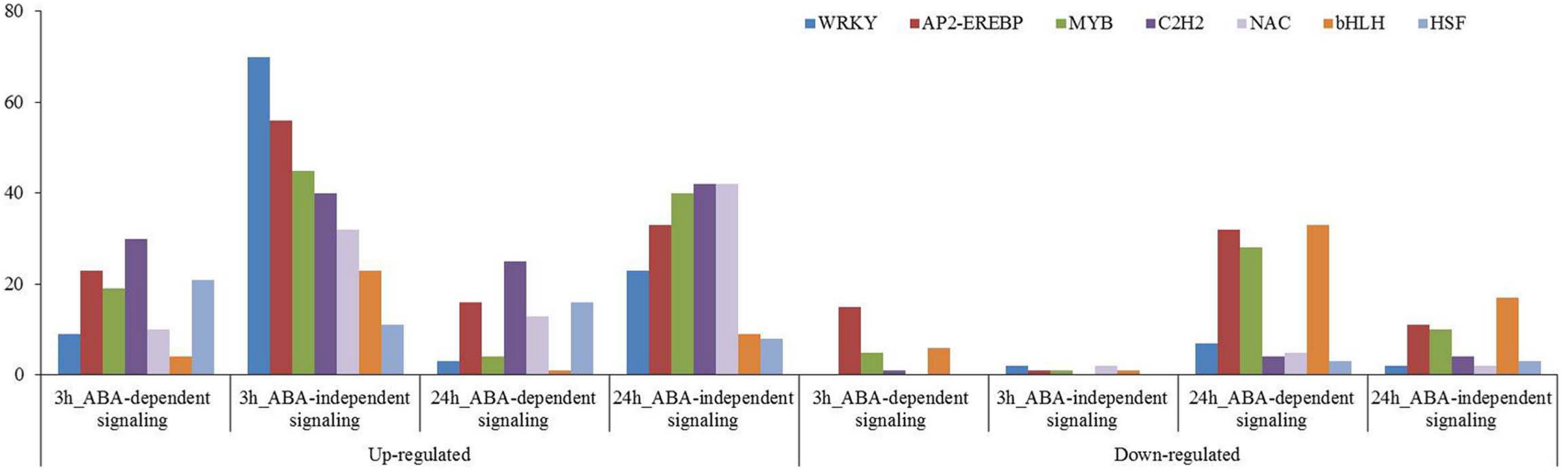
Transcription factors in response to osmotic stress at 3 and 24 h. Those involved in ABA-dependent and -independent pathways in response to osmotic stress are summarised.

Many transcription factors have been reported to participate in plant osmotic-stress response. In this study, 563 up-regulated and 69 down-regulated transcription factors were identified for the 3 h osmotic stress treatment, and 432 up-regulated and 404 down-regulated transcription factors were identified for the 24 h osmotic stress treatment. Among them, 431, including 403 up-regulated and 28 down-regulated, and 420, including 306 up-regulated and 114 down-regulated, transcription factors were found in ABA-independent pathway at 3 and 24 h osmotic stress, respectively. Two hundred and one, including 160 up-regulated and 41 down-regulated, and 416, including 126 up-regulated and 290 down-regulated, transcription factors were found in ABA-dependent pathway at 3 and 24 h osmotic stress, respectively (Supplementary File 3). There were more up-regulated transcription factors that are involved in the ABA-independent pathway than that in the ABA-dependent pathway at 3 and 24 h; conversely, more down-regulated transcription factors were found in the ABA-dependent pathway than that in the ABA-independent pathway. These transcription factors, differentially expressed under osmotic stress, were classified into 55 types, and 28 types were identified in the ABA-dependent pathway at 3 h, followed by 42 at 24 h (Supplementary File 3). WRKY, AP2-EREBP, MYB, C2H2, NAC, bHLH, and HSF were significantly expressed under osmotic stress, and the number of these transcription factors was all more than 50 (Fig. 7). These transcription factors should play important roles in response to osmotic stress in wheat roots.

## Discussion

Osmotic stress is one of the main abiotic stress factors that limits plant growth and damages normal physiological functions (Lee & Mudge, 2013). Previous studies showed that osmotic-responsive genes in plants play their roles mainly through ABA-dependent and -independent pathways (Yoshida, Mogami & Yamaguchi-Shinozaki, 2014; Agarwal & Jha, 2010). Although many studies have investigated the main molecular mechanisms underlying both pathways, osmotic-related genes in the ABA-dependent or -independent pathways in wheat have not yet been integrally identified. In this study, osmotic-responsive genes in bread wheat were divided into ABA-dependent and -independent pathways. The results showed that 1,859 and 3,734 genes participate in the ABA-dependent and -independent pathways at 3 h osmotic stress, and 7,436 and 5,129 genes participate in the ABA-dependent and -independent pathways at 24 h osmotic stress, respectively. According to Mapman analysis, most of the genes involved in ABA biosynthesis and ABA signalling participate in the ABA-dependent pathway, indicating that this classification system is reasonable.

Glutathione is an antioxidant that protects SH-group in proteins from oxidation to maintain properties of proteins and enzymes (Noctor et al., 2012). In this study, 53 and 77 *GSTs* in the GSH metabolism pathway were identified at 3 and 24 h of osmotic stress, respectively. Among them, 31 and 38 *GST*s in the ABA-independent pathway were observed at 3 and 24 h of osmotic stress, and there were 22 and 39 *GST*s in the ABA-dependent pathway at 3 and 24 h of osmotic stress, respectively. In our previous proteomic profiling study, we found ten proteins involved in GSH metabolism in response to osmotic stress. Of these, eight were identified as GSTs (Ma et al., 2016). Other studies have also shown that *GSTs* play important roles in drought stress response of wheat (Choi, An & An, 2008; Gallé et al., 2009; Chen et al., 2012). Combined with the results of previous studies, we confirm that *GSTs* are essential for osmotic-stress response in wheat. Therefore, further systematic investigation is warranted.

Researches have shown that ABA can generally enhance drought tolerance in plants (Zhang et al., 2012; Xu et al., 2015; Bano, Ullah & Nosheen, 2012), and recent studies indicate that PP2C and PYR/PYL proteins form the central core of ABA signal transduction (Dalal & Inupakutika, 2014). In the present study, transcriptome analysis revealed that 15 and 27 genes were involved in the ABA signalling at 3 and 24 h of osmotic stress, respectively. The majority of them were classified into the ABA-dependent pathway, indicating that this classification is reasonable. One and 11 *PYR/PYLs* and 14 and 16 *PP2Cs* were found at 3 and 24 h of osmotic stress, respectively. These results suggest that *PYR/PYLs* and *PP2Cs* are important osmotic-stress response components in ABA signalling. We found that eleven *PYR/PYLs* were down-regulated in response to osmotic stress, which corroborates the data reported by Kalapos et al. (2016), whereas all *PP2Cs* were up-regulated in response to osmotic stress. Therefore, a more profound understanding of the interaction between *PP2Cs* and *PYR/PYLs* will help in elucidating the molecular mechanisms of plant adaptations to stress. A close relationship between ABA signalling and calcium ions signalling exists (Edel & Kudla, 2016), and we found that osmotic stress first activate *Rbohs* causing the accumulation of ROS. There is a negative relationship between the process of ROS accumulation and PP2Cs that were involved in ABA signalling (Mittler & Blumwald, 2015), and we found that this process was inhibited with increasing ABA content. Therefore, we speculate that ABA should serve as a negative regulatory factor of *Rbohs.* From 3 to 24 h osmotic stress, many *CaM/CMLs*, which would further induce expression of nitric-oxide (NO) synthase for NO production, exhibited up-regulated; therefore, NO should be important for regulating the response to osmotic stress via calcium ions signalling in wheat root.

**Figure 8:**
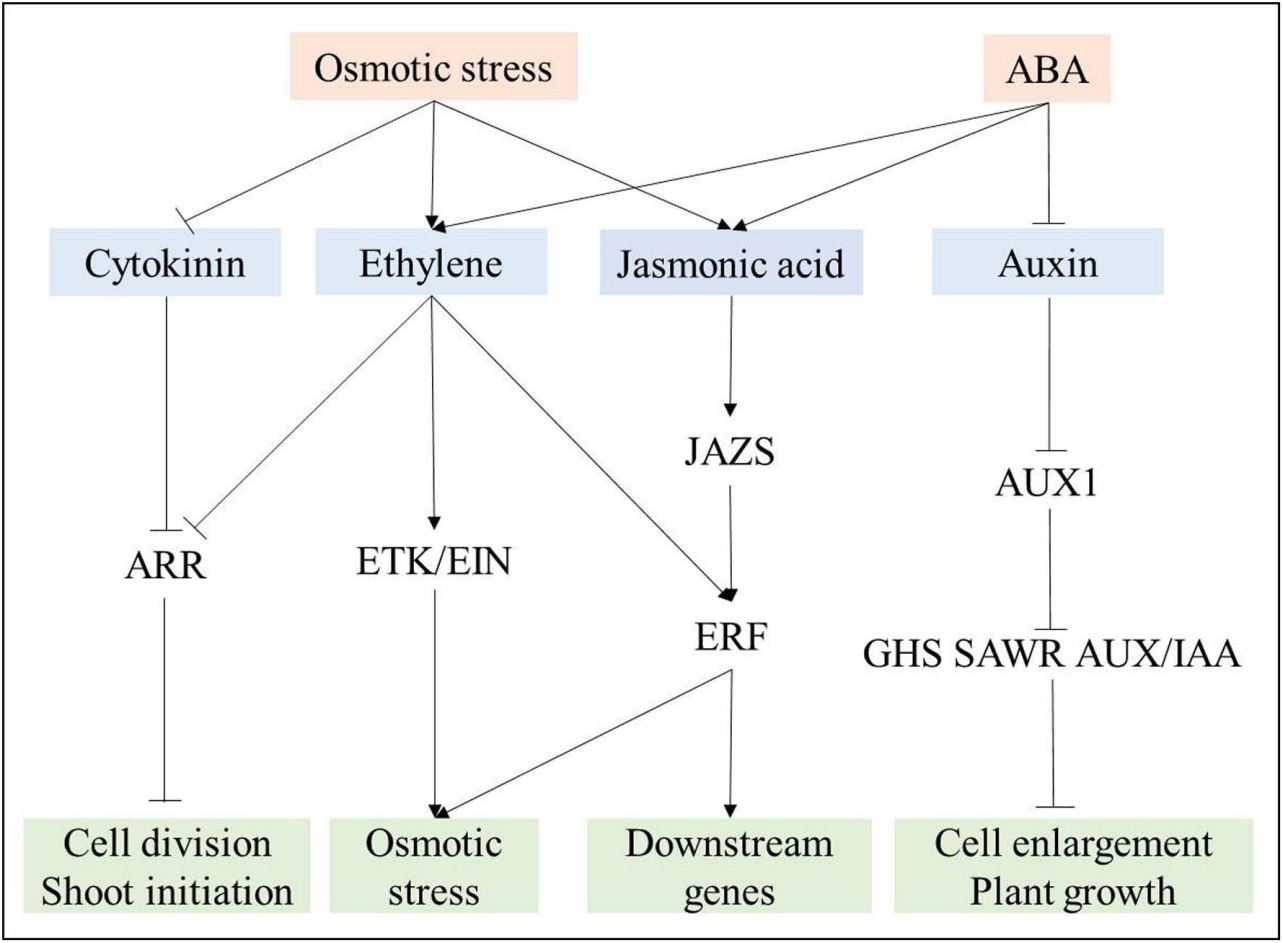
Interaction among phytohormones signal transduction responding to osmotic stress. Positive and negative regulatory actions are indicated by arrows and lines with bars, respectively.

Based on the analysis of phytohormones signal transduction in response to osmotic stress, a complex relationship among these phytohormones was revealed (Fig. 8). Cytokinin and auxin signal transduction were negatively regulated by osmotic stress and ABA, respectively (Fig. 8), further suppressing root growth. Ethylene and jasmonic acid signal transduction were induced by both osmotic stress and ABA. Ethylene further suppressed ARR, which participates in cytokinin signal transduction, and induced ETR, EIN, and ERF to regulate downstream genes for the response to osmotic stress (Kazan, 2015). As JAZs are located upstream of ERF (Kazan, 2015), ERF also plays its role via jasmonic acid signal transduction (Fig. 8). Overall, many DEGs are involved in phytohormones signal transduction, and a complex relationship among osmotic-responsive genes depending on phytohormones signal transduction likely exists.

## Conclusion

This present study primarily focused on ABA-dependent and -independent pathways in response to osmotic stress in bread wheat. Comprehensive investigations of important metabolic processes were conducted, and many candidate genes were identified. Detailed analyses of the pathways elucidated in our study would deepen our understanding of the molecular mechanism of osmotic-resistance in wheat. They would also provide numerous candidate osmotic-responsive genes to use for subsequent investigations.

### Supplemental Information

10.7717/peerj.6519/supp-1 Supplemental Information 1 File S1. Primers for qRT-PCR and Correlations between RNA-seq and qRT-PCR.

10.7717/peerj.6519/supp-2 Supplemental Information 2 File S2. Derailed information of differentially expressed genes responding to osmotic stress.

10.7717/peerj.6519/supp-3 Supplemental Information 3 File S3. The number of differentially expressed transcription factors in ABA-dependent and ABA-independent pathways responding to osmotic stress after 3 h and 24 h.

## Additional Information and Declarations

### Data Availability

The following information was supplied regarding data availability: The raw data are available in the Supplemental Files.
